# Expression Profiling Identifies the Noncoding Processed Transcript of HNRNPU with Proliferative Properties in Pancreatic Ductal Adenocarcinoma

**DOI:** 10.3390/ncrna3030024

**Published:** 2017-08-25

**Authors:** Dhruvitkumar S. Sutaria, Jinmai Jiang, Ana Clara P. Azevedo-Pouly, Eun Joo Lee, Megan R. Lerner, Daniel J. Brackett, Jo Vandesompele, Pieter Mestdagh, Thomas D. Schmittgen

**Affiliations:** 1 Department of Pharmaceutics, College of Pharmacy, University of Florida Gainesville, Gainesville, FL 32608, USA; dsutaria@ufl.edu (D.S.); JJiang@cop.ufl.edu (J.J.); 2 Department of Pharmaceutics and Pharmaceutical Chemistry, College of Pharmacy, Ohio State University Columbus, Columbus, OH 43210, USA; ana.azevedo-pouly@utah.edu; 3 College of Pharmacy and Wonkwang Oriental Medicines Research Institute, Wonkwang University, 54538 Iksan, Korea; eundoo@gmail.com; 4 Department of Surgery, University of Oklahoma Heath Science Center, Oklahoma City, OK 73104, USA; Megan-Lerner@ouhsc.edu (M.L.); Daniel-Brackett@ouhsc.edu (D.B.); 5 Center for Medical Genetics, Ghent University Hospital, 9000 Ghent, Belgium; joke.vandesompele@ugent.be (J.V.); Pieter.Mestdagh@UGent.be (P.M.)

**Keywords:** pancreatic cancer, HNRNPU processed transcript, ncRNA00201, HNRNPU, lncRNA, LNA^TM^ gapmers

## Abstract

A gene array was used to profile the expression of 22,875 long non-coding RNAs (lncRNAs) and a large number of protein coding genes in 47 specimens of pancreatic ductal adenocarcinoma (PDAC), adjacent benign pancreas and the pancreas from patients without pancreatic disease. Of the lncRNAs profiled, the expression of 126 were significantly increased and 260 were decreased in the tumors (*p* < 0.05, 2-fold). The expression of one lncRNA in particular, heterogeneous nuclear ribonucleoprotein U (HNRNPU) processed transcript (also known as ncRNA00201) was among the most significantly deregulated (increased four-fold) in the tumors compared to normal/adjacent benign tissues. Increased expression of HNRNPU processed transcript was associated with poor prognosis for patients with PDAC. The expression of HNRNPU processed transcript was increased in PDAC cell lines compared to noncancerous pancreatic cell lines. LNA^TM^ gapmer mediated inhibition of HNRNPU processed transcript reduced cell proliferation in Patu-T and PL45 pancreatic cancer cell lines. Reduced invasion and migration was reported upon HNRNPU processed transcript knockdown in Patu-T cells. Small interfering RNA (siRNA) knockdown of the HNRNPU protein coding gene correlated with a 55% reduction in the HNRNPU processed transcript expression and a corresponding reduction in proliferation of Patu-T and PL45 cells. However, gapmer inhibition of HNRNPU processed transcript did not affect HNRNPU mRNA levels. The lncRNA HNRNPU processed transcript expression is increased in both PDAC tissues and cell lines; knockdown of this lncRNA further reduces proliferation and invasion/migration of pancreatic carcinoma cells.

## 1. Introduction

Pancreatic ductal adenocarcinoma (PDAC) is the fourth most lethal cancer in the USA and it is estimated that it will become the second most deadly cancer in the country by 2030 [1,2]. Invasive PDAC develops via low-grade to high-grade precursor lesions known as pancreatic intraepithelial neoplasia (PanIN) [3]. PanIN-2 is typically proceeded by activating mutations in KRAS, an oncogene that is mutated in greater than 90% of PDAC. In addition to KRAS activation, mutations in tumor suppressors—including CDKN2A, SMAD4, and TP53—are commonly present in the tumors and likely contribute to the development of invasive PDAC in patients [4].

While mutations in protein coding genes undoubtedly initiate and promote PDAC, it is possible that noncoding RNAs (ncRNA) play a causative role as well. Numerous profiling and sequencing studies over the past decade have demonstrated that ncRNAs are differentially expressed in PDAC. We and others have shown that microRNAs (miRNAs) have unique expression patterns in PDAC specimens [5,6,7]. Additional profiling of long non-coding RNAs (lncRNAs) [8,9], circular RNAs [10] and transcribed ultraconserved RNAs [11] in PDAC tissues demonstrate that levels of noncoding transcripts differ between PDAC and normal/adjacent benign pancreas. In addition to differential expression, ncRNAs exhibit functionality in PDAC. H19 is overexpressed in pancreatic cancer and promotes the epithelial-mesenchymal transition by reducing expression levels of the let-7 tumor suppressor [12]. HOX transcript antisense intergenic RNA (HOTAIR) was overexpressed in PDAC and HOTAIR knockdown in PDAC cells inhibited tumor growth in a mouse xenograft model suggesting a pro-oncogenic function for this lncRNA [13]. The lncRNA MALAT1 advanced progression of tumors in vivo via activating autophagy in PDAC [14].

Our goal here was to discover new lncRNA transcripts and study their functionality in PDAC. We used a custom cDNA microarray platform to profile the expression of 22,875 lncRNAs. A number of lncRNAs were differentially expressed in patients’ specimens. We focused our attention on one lncRNA transcript in particular, heterogeneous nuclear ribonucleoprotein U (HNRNPU, also known as ncRNA00201) processed transcript, as it was one of the most highly deregulated lncRNAs in PDAC. Knockdown of HNRNPU processed transcript reduces proliferation and in vitro migration/invasion of PDAC cell lines.

## 2. Results

### 2.1. LncRNAs Are Differentially Expressed in Pancreatic Ductal Adenocarcinoma

**Figure 1:**
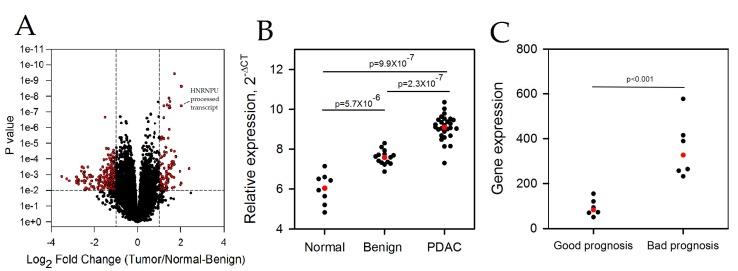
(**A**) Expression values of 22,875 lncRNAs in the PDAC and normal-benign pancreas were converted to Log_2_ (fold change) and were compared to *p*-values using a volcano plot. A threshold of *p* < 0.01 and fold change greater than two-fold (red symbols) was applied to determine statistical significance. The data for heterogeneous nuclear ribonucleoprotein U (HNRNPU) processed transcript is highlighted. (**B**) Expression of the HNRNPU processed transcript expression in PDAC, adjacent benign tissue and normal pancreas from the cDNA microarray. All three comparisons (normal vs. benign; normal vs. tumor and benign vs. tumor) were significant at *p* < 0.001 (Tukey’s multiple comparison test). (**C**) Data from Gene expression omnibus (GEO) profile GSE42952 were mined to present the HNRNPU processed transcript expression in those PDAC patients with good or poor prognosis. Red symbols, median values.

The expression of 22,875 lncRNAs was profiled in a cohort of 48 pancreas specimens (8 normal, 15 adjacent benign, and 25 PDAC). Of the lncRNAs profiled, the expression of 126 were significantly increased and 260 were decreased in the tumors (false discovery rate (FDR) *p* < 0.05; Fold change two-fold). The volcano plot generated shows all differentially expressed lncRNAs between the normal-benign and PDAC patient sample groups (Figure 1A). The expression of several lncRNA’s sequentially increased or decreased in the PDAC to normal-benign pancreas comparison. Differentially expressed lncRNAs were ranked based on the –log(p) × Fold Change and the top 10 ranked lncRNAs are presented in Table 1. 

HNRNPU processed transcript was of particular interest as it was one of the top four differentially expressed lncRNAs. The expression of the HNRNPU processed transcript significantly increased in the PDAC compared to the normal and benign specimens (Figure 1B). qPCR confirmation of the array data held for the normal versus tumor (*p* < 0.001), normal versus benign (*p* < 0.05), and for the tumor versus normal-benign (*p* < 0.01) comparisons but not for the benign versus tumor comparison (*p* > 0.05). Data mining of publically available gene profiling results further showed that HNRNPU processed transcript lncRNA expression is increased in patients with poor prognosis compared to those with good prognosis [15] (Figure 1C). We also mined the most recent version of the TCGA metadata for PDAC to determine if HNRNPU processed transcript correlated with patient survival, however the result was not statistically significant.

### 2.2. HNRNPU Processed Transcript Expression Is Increased in Pancreatic Cancer Cell Lines

**Figure 2:**
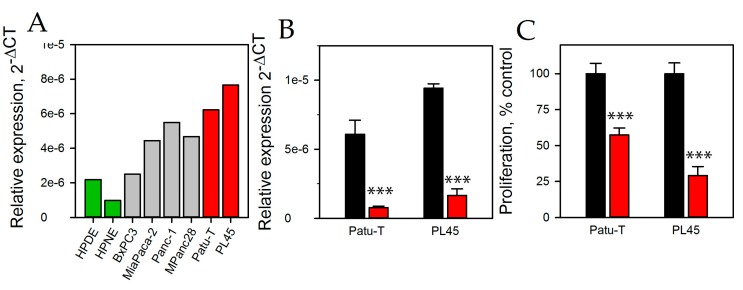
(**A**) qPCR was used to determine the relative expression of HNRNPU processed transcript in immortalized pancreas epithelial cell lines (green bars) and PDAC cell lines (grey and red bars). Patu-T and PL45 cells (red bars) were used in the remainder of the study. (**B**) Relative expression of HNRNPU processed transcript as determined by qPCR in Patu-T and PL45 cells following a 48 h exposure of 100 nM of control LNA^TM^ gapmer (black bars) or LNA gapmer to HNRNPU processed transcript (red bars). Data were normalized to 18 S rRNA. (**C**) Cell proliferation was determined using a WST1 assay in Patu-T and PL45 cells following a 48 h exposure of 100 nM of control LNA gapmer (black bars) or LNA gapmer to HNRNPU processed transcript (red bars). *** *p* < 0.001.

In order to perform functional analyses, the expression of HNRNPU processed transcript was determined in pancreatic cancer cell lines. The expression of HNRNPU processed transcript was three- to four-fold higher in several of the PDAC cell lines compared to the normal pancreatic epithelial cells (Figure 2A). The two PDAC cell lines with the highest expression of HNRNPU processed transcript, Patu-T and PL45, were used for the remainder of the study. PL45 was derived from a primary, poorly differentiated PDAC. The cell line has the G12D KRAS mutation and a codon 225 p53 mutation. Patu-T is derived from a well differentiated liver metastasis of a PDAC. It has the KRAS G12V mutation and a missense mutation in p53. To evaluate a functional role of HNRNPU processed transcript in PDAC, its expression was inhibited using LNA gapmers. As the location of many lncRNAs is nuclear, we used LNA gapmers instead of siRNA, as gapmers are functional in the nucleus as well as the cytoplasm. Successful RNA knockdown was achieved; LNA gapmers reduced the HNRNPU processed transcript expression by 90% and 83% in Patu-T and PL45 cells, respectively (Figure 2B). HNRNPU processed transcript knockdown reduced cell proliferation by 42% in Patu-T cells and 68% in PL45 cells (Figure 2C).

**Figure 3:**
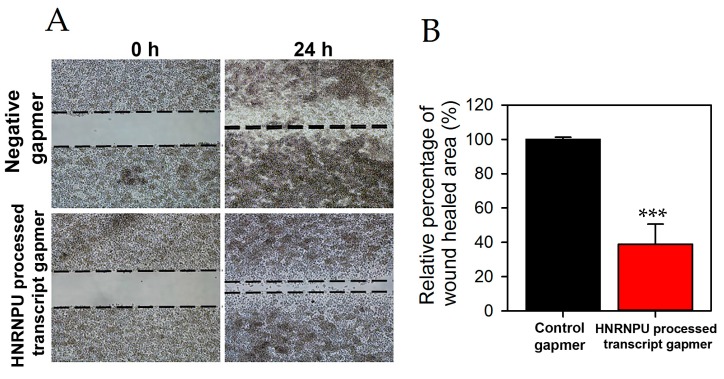

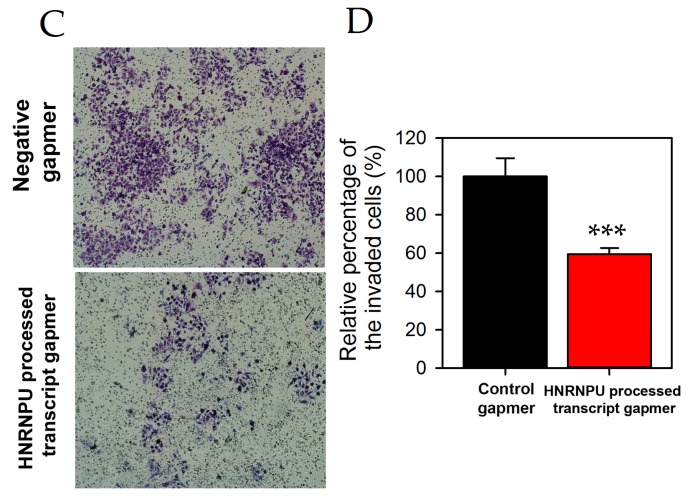
Patu-T cells were treated with 100 nM control or HNRNPU processed transcript gapmer. (**A**,**B**) Cell migration assay was measured at time zero or 24 h later as described in Materials and Methods. Wound healed area determined from 4 × magnification images. (**C**,**D**). Boyden chamber invasion assays were conducted with control and HNRNPU processed transcript gapmer transfected Patu-T cells. The number of the invading cells was determined from five different fields for each experiment. *** *p* < 0.001.

Next, we determined whether HNRNPU processed transcript knockdown could inhibit in vitro cell migration and invasion. Patu-T cells were transfected with lncRNA gapmer under the identical conditions shown to suppress HNRNPU processed transcript expression. Negative control gapmer was used as a control for both the assays. Wound healing was significantly reduced in Patu-T cells after lncRNA knockdown (Figure 3A,B). In addition, cell invasiveness was also significantly reduced following lncRNA knockdown (Figure 3C,D). 

### 2.3. HNRNPU Processed Transcript Expression Is Regulated by HNRNPU Protein Coding Gene

**Figure 4:**
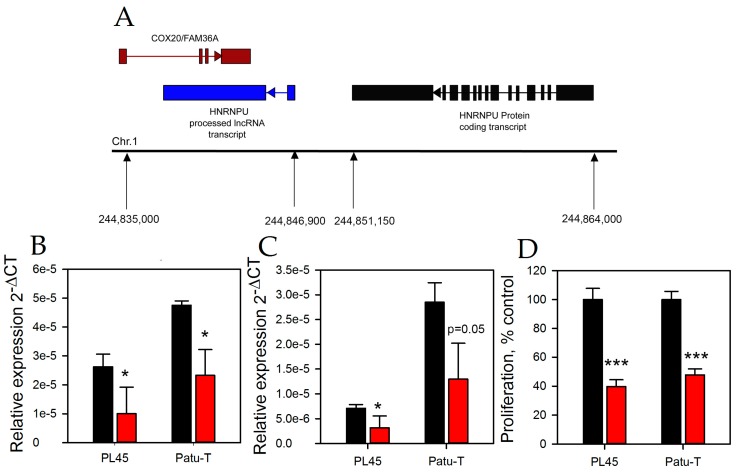
(**A**) Map of genomic location of the HNRNPU processed transcript lncRNA and HNRNPU and COX20/FAM36A protein coding genes. (**B**) Patu-T and PL45 cells were exposed to 50 nM of HNRNPU siRNA (red bars) or control oligo (black bars) for 48 h. The expression of HNRNPU protein coding gene relative to 18S rRNA was determined by qPCR. (**C**) Patu-T and PL45 cells were exposed to 50 nM of HNRNPU siRNA (red bars) or control oligo (black bars) for 96 h and the expression of HNRNPU processed transcript relative to 18S rRNA was deterined by qPCR. (**D**) Cell proliferation was measured using a WST1 assay in the cell lines treated with HNRNPU siRNA (red bars) or control oligo (black bars) for 96 h. * *p* < 0.05, *** *p* < 0.001.

HNRNPU is a protein coding gene that belongs to the class of heterogenous ribonucleoprotein RNA binding proteins. The HNRNPU processed transcript gene is located about 4 kbp adjacent to HNRNPU; HNRNPU processed transcript lies antisense to the COX20/FAM36A gene (Figure 4A). In an effort to link HNRNPU to HNRNPU processed transcript, the HNRNPU processed transcript expression was determined after knocking down the HNRNPU mRNA using siRNA. Successful knockdown of HNRNPU (Figure 4B) correlated with a 55% reduction in the HNRNPU processed transcript expression in both PL45 and Patu-T cells (Figure 4C). These data suggest that the RNA binding protein directly or indirectly regulates the expression of HNRNPU processed transcript. Since a reduction in HNRNPU mRNA positively correlated with a decrease in the HNRNPU processed transcript, we next asked if reduction in HNRNPU expression also reduced proliferation. Similar to the HNRNPU processed transcript knockdown experiment, HNRNPU inhibition reduced Patu-T and PL45 cell proliferation by approximately 60% and 53%, respectively (Figure 4D). The HNRNPU mRNA was measured in both Patu-T and PL45 cells following inhibition of HNRNPU processed transcript but no significant change in the HNRNPU mRNA was observed (FC, gapmer/control = 1.3, *p* = 0.48 for Patu-T cells and FC, gapmer/control = 0.64, *p* = 0.06 for PL45 cells). Since the HNRNPU processed transcript gene lies antisense to COX20 (Figure 4A), we also measured COX20 mRNA in both the HNRNPU processed transcript and HNRNPU knockdown experiments. However, COX20 levels were unchanged with knockdown of either genes (FC, gapmer/control = 0.94, *p* = 0.65 for Patu-T cells and FC, gapmer/control = 1.06, *p* = 0.64 for PL45 cells). 

## 3. Discussion

An increasing number of novel lncRNA transcripts have been reported, highlighting the important role of lncRNAs in cell biology, development, and disease [16,17]. LncRNAs continue to generate interest in the field of oncology due to their functional involvement in regulating cancer growth and malignant transformation [18,19,20]. We report here a completely unexplored lncRNA in cancer, which is increased in human PDAC tissues and PDAC cell lines. HNRNPU processed transcript, also known as C1orf199/ncRNA00201, is a processed transcript of the HNRNPU gene located on chromosome 1q44. LncRNAs are denoted as processed transcripts when they do not retain the intronic sequences of protein coding genes and lack an open reading frame. While we are unaware of the mechanism by which HNRNPU processed transcript is transcribed from the HNRNPU transcript, it is clear from the genomic map that both the processed and protein coding transcripts do not overlap with each other and would thus presumably behave differently (Figure 4A). To further confirm a lack of coding potential of HNRNPU processed transcript, we used the coding potential calculator (CPC) program [21]. This program provided a coding potential score of −0.98372 which is considered a weak protein coding potential. HNRNPU belongs to the class of RNA binding proteins which has been shown to regulate pre-mRNA processing in the nucleus [22]. Apart from processing, they also play a role in mRNA metabolism and transport [23]. HNRNPU directly binds to WT1 lncRNA and thereby modulates its transcriptional activation [24]. Another study showed that lncRNA FIRRE interacts directly with HNRNPU through a 156-bp repeating domain, enabling the lncRNA to maintain its multichromosomal nuclear interactions [25].

One means of increasing lncRNA expression in cancer is the stabilization of the ncRNA following direct binding to RNA binding proteins [26]. For example, HuR RNA binding protein stabilizes the expression of Neat1 lncRNA in ovarian cancer tissues through direct binding [27]. One mechanism by which RNA binding proteins may protect the lncRNA transcript from degradation is to shuttle the lncRNA between nucleus and cytoplasm offering protection from nuclease degradation [28,29]. We show here that siRNA knockdown of the HNRNPU protein coding mRNA results in reduction of its processed lncRNA transcript (i.e., HNRNPU processed transcript). This implies that the HNRNPU RNA binding protein interacts with HNRNPU processed transcript, preventing it from cellular degradation. 

Knockdown of HNRNPU processed transcript using LNA gapmers decreased cell proliferation, migration, and invasion of Patu-T pancreatic cell line, suggesting HNRNPU processed transcript plays a role in cell proliferation and invasion. In conclusion, we present that HNRNPU processed transcript lncRNA is upregulated in PDAC tissues and cell lines, and regulates in vitro proliferation, invasion, and migration.

## 4. Materials and Methods 

### 4.1. Patient Tissue Specimens

A total of 15 adjacent benign tissues and 24 flash frozen primary PDAC tissues were collected from patients who underwent surgical resection at the University of Oklahoma Health Sciences Center under an approved protocol from the institution’s IRB. Specimens were separated into tumor and benign tissues under the supervision of a pathologist. Eight normal pancreas tissues were acquired from various suppliers obtained from subjects that expired from causes other than pancreatic cancer. RNA isolation was carried out from the flash frozen tissues by pulverization method using Trizol method (Thermo Scientific, Waltham, MA, USA). RNA samples with RNA integrity number (RIN) of 6.0 or higher was used for analysis. Patient demographics were previously listed in Jiang et al., [11].

### 4.2. Microarray Data Processing and Analysis

A 54,000 gene expression Agilent array (Agilent, Santa Clara, CA, USA- MicroArray Design ID 041648) containing probes for 22,875 lncRNA transcripts was used in the study. To determine the most significantly expressed lncRNAs, the expression values were converted to Log_2_ (fold change tumor/normal pancreas) and the Log_2_ (fold change) are compared to *p*-values using a volcano plot. Dots represent mean independent biological replicates for a given lncRNA. A threshold of *p* < 0.01 and fold change greater than two-fold was applied to determine significance; and −Log (*p*) × Fold Change calculation was used to identify lncRNAs with increased and decreased expression levels. All raw gene expression data are presented at GEO profile GSE91035.

### 4.3. Cell Culture and Treatments

Human pancreatic cancer cell lines Panc1, MiaPaCa-2, BxPC3, and PL45 were obtained from American Type Culture Collection (Manassas, VA, USA) or from other investigators. The HPDE cell line was obtained from Dr. Ming-Sound Tsao, Ontario Cancer Institute, Toronto, Ontario. HPDE cells were maintained in keratinocyte medium containing bovine pituitary extract and human rEGF (Invitrogen, Waltham, MA, USA) at 30 µg/mL and 0.2 ng/mL concentrations respectively. HPNE cells were obtained from Dr. Michel Ouellette at University of Nebraska. Patu-T pancreatic cancer cells were generously provided to us by Dr. Irma Van Die at Department of Molecular Cell Biology and Immunology, VU University Medical Centre, Amsterdam, The Netherlands. Patu-T, Panc-1, MiaPaCa-2, and PL45 cells were maintained in Dulbecco’s modified eagle’s medium (ATCC) supplemented with 10% fetal bovine serum (Sigma Aldrich, St. Louis, MO, USA). All the cell lines were successfully authenticated as previously mentioned [11].

The siRNAs targeting the HNRNPU gene and the negative control were purchased from Thermo Scientific (Waltham, MA, USA). LncRNA gapmers targeting HNRNPU processed transcript was custom prepared as LNA gapmers (Exiqon, Vedbaek, Denmark). The sequences for the four LNA gapmers used during the study are: negative control A: 5′-AACACGTCTATACGC-3′; HNRNPU processed transcript_GP1: 5′-GTAGTAGTCATCTGTA-3′; HNRNPU processed transcript_GP2: 5′-AAGTTAGCGGCAGATC-3′; and HNRNPU processed transcript_GP3: 5′-GCACAGAAATCGAGCA-3′. For the LncRNA knockdown evaluation, Patu-T and PL45 cells were plated at a seeding density of 1.5 × 10^5^ and 2 × 10^5^ cells, respectively. The cells were then transfected with 100 nM of LNA gapmers using Lipofectamine 3000 and incubated for 48 h. RNA isolation was then carried out using RNAeasy kit (Qiagen, Germantown, MD, USA) HNRNPU siRNA transfections were carried out at 50 nM siRNA concentrations in Patu-T and PL45 cells with the same conditions mentioned earlier.

### 4.4. qPCR

cDNA was generated from patient and cell culture samples using random primers and superscript II reverse transcriptase enzyme (Thermo Scientific, Waltham, MA, USA). qPCR for the HNRNPU processed transcript was performed using gene specific RT primers as previously described [30]. 18S rRNA was used as the reference gene and data were analyzed using the 2^−ΔCT^ method. 

### 4.5. Cell Proliferation Assay

PL45 and Patu-T cells were plated at a seeding density of 4000 and 2500 cells per well, respectively. One hundred nM oligo was transfected into the cells the following day and incubated for 96 h. Cell proliferation was evaluated using a WST1 assay (Roche, Indianapolis, IN, USA).

### 4.6. Migration and Invasion Assay

Invasion and migration assays were carried out on Patu-T cells using 24 cell well invasion assays, basement membrane (Cell Biolabs Inc., San Diego, CA, USA) and culture inserts 2 well in µ-Dish 35 mm (Ibidi, Fitchburg, WI, USA). The cells were transfected in a similar manner to the knockdown experiment and incubated for 48 h. Cells were trypsinized and resuspended at two different concentrations 3 × 10 ^5^ cells/ mL and 1 × 10 ^6^ cells/ mL for migration and invasion assay, respectively. The two assays were performed according to the manufacturer’s protocol. 

### 4.7. Statistical Analysis

Error bars presented on all graphs are standard deviation of the mean. All two sample comparisons were performed using a two-tailed Student’s *t*-test. Tukey’s test was performed using GraphPad Prism software (La Jolla, CA, USA). The equation −log (*p*) × FC was used to rank the most significant, differentially expressed lncRNA; where *p* is the FDR *p*-value and FC is the absolute value of the fold change. *p*-values were FDR corrected using the Benjamini & Hochberg approach [31] using R. Gene omnibus data set GSE42952 was mined to examine the correlation between ncRNA00201 expression and patient prognosis. As described in [15], a good prognosis was defined as overall survival and disease-free survival >50 months and a bad prognosis as overall survival <19 months and disease-free survival <7 months.

## Figures and Tables

**Table 1:** Most significant, differentially expressed lncRNAs.

Gene Symbol	Fold Change	FDR	−log(*p*) × Fold Change
ENST00000407852	4.09	2.37 × 10^−9^	35.2
TCONS_00012350	−11.4	0.001301	32.9
NCRNA00265	3.27	3.63 × 10^−10^	30.9
NCRNA00201	4.08	4.03 × 10^−8^	30.1
ENST00000536141	−9.73	0.001841	26.6
ENST00000543206	−8.94	0.002479	23.3
ENST00000452840	2.83	2.20 × 10^−8^	21.7
TCONS_00022114	2.75	1.32 × 10^−8^	21.7
ENST00000558097	−5.94	0.000323	20.7
ENST00000415104	2.80	4.03 × 10^−8^	20.7
